# Evaluation of resazurin-based assay for rapid detection of polymyxin-resistant gram-negative bacteria

**DOI:** 10.1186/s12866-019-1692-3

**Published:** 2020-01-08

**Authors:** Huaiyu Jia, Renchi Fang, Jie Lin, Xuebin Tian, Yajie Zhao, Lijiang Chen, Jianming Cao, Tieli Zhou

**Affiliations:** 1 Department of Clinical Laboratory, the First Affiliated Hospital of Wenzhou Medical University, Wenzhou, Zhejiang province China; 2 School of Laboratory Medicine and Life Sciences, Wenzhou Medical University, Wenzhou, Zhejiang Province China

**Keywords:** Rapid ResaPolymyxin *Acinetobacter*/*Pseudomonas* NP test, Colistin-resistant, Gram-negative bacteria, Rapid diagnosis

## Abstract

**Background:**

Colistin resistance is considered a serious problem due to a lack of alternative antibiotics. The Rapid ResaPolymyxin *Acinetobacter*/*Pseudomonas* NP test is a resazurin reduction-based technique that relies on the visual detection of bacterial growth in the presence of a defined concentration of colistin. The aim of this study was to evaluate the performance of the Rapid ResaPolymyxin *Acinetobacter*/*Pseudomonas* NP test in the detection of colistin susceptibility in common clinical Gram-negative bacteria.

**Results:**

A total of 253 clinical isolates from a teaching hospital, including *Acinetobacter baumanii* (*n* = 58, 8 colistin-resistant), *Pseudomonas aeruginosa* (*n* = 61, 11 colistin-resistant), *Klebsiella pneumoniae* (*n* = 70, 20 colistin-resistant) and *Escherichia coli* (*n* = 64, 14 colistin-resistant) were tested in this study. The sensitivity and specificity of the Rapid ResaPolymyxin *Acinetobacter*/*Pseudomonas* NP test compared to Broth microdilution method was 100 and 99%, respectively.

**Conclusions:**

Our results suggest that Rapid ResaPolymyxin *Acinetobacter*/*Pseudomonas* NP test could be used as an accurate detection method for colistin resistance.

## Background

Polymyxin E, also known as colistin is a multicomponent polypeptide antibiotic, which belongs to the group of polymyxin [1]. Polymyxin E was discovered in the 1940s; yet, later on, it was abandoned in clinical practice due to its increased nephrotoxicity. However, due to the increase of multidrug resistance (MDR) in Gram-negative bacteria, especially in the ESKAPE pathogens (*Enterococcus faecium*, *Staphylococcus aureus*, *Klebsiella pneumoniae*, *Acinetobacter baumannii*, *Pseudomonas aeruginosa*, and *Enterobacter* species), colistin has been applied in clinical practice for the last few years as the last resort treatment option [2, 3]. Currently, colistin resistance is considered a serious problem, due to a lack of alternative antibiotics [4, 5]. As for now, rapid identification of colistin resistance is considered essential for the effective control of MDR Gram-negative bacteria infection.

Broth microdilution (BMD) is the only reference method that has been recommended by the European Committee on Antimicrobial Susceptibility Testing (EUCAST) and Clinical and Laboratory Standards Institute (CLSI) for the detection of minimum inhibitory concentrations (MICs) of colistin [6, 7]. Nevertheless, colistin antimicrobial susceptibility testing is very challenging to perform [8, 9]. For example, the operational steps of BMD are complex and time-consuming, making it unsuitable for clinical use [10]. Clinical microbiology laboratories are especially affected by the lack of an accurate, fast and easy-to-conduct method to test the colistin susceptibility [11–13]. Therefore, it is of great significance for clinical anti-infective treatment to develop and promote new, convenient, economical, rapid and accurate colistin sensitivity detection method.

In 2016, Nordmann et al developed the Rapid Polymyxins NP test for *Enterobacteriaceae spp* [14]. The method can be used to detect bacteria that can grow, metabolize glucose, and produce acid in the presence of polymyxin such as polymyxin B or colistin through color changes of PH indicators. However, one of the significant limitations when using this approach is that it cannot be applied for non-fermentative bacteria such as *A. baumannii* and *P. aeruginosa*. More recently, Lescat et al have developed a rapid resazurin-mucoid susceptibility test method called Rapid ResaPolymyxin *Acinetobacter*/*Pseudomonas* NP test, which can quickly detect the sensitivity of colistin for both *Enterobacteriaceae spp* and non-fermentative bacteria within 4 h [15]. The method is mainly based on detection of the strain viability by observing the color change of resazurin (an active colorant) from blue to purple or pink in the presence of colistin (3.75 mg/L).

In this study, we analyzed the performance of the Rapid ResaPolymyxin *Acinetobacter*/*Pseudomonas* NP test in the detection of colistin susceptibility in 253 nonduplicate clinical Gram-negative isolates aiming to provide a basis for the popularization and application of a new method for rapid screening of colistin-resistant common clinical Gram-negative bacteria.

## Results

The colistin MICs of the 253 Gram-negative isolates ranged from ≤0.06 to ≥32 mg/L. BMD results were used as a standard, and 53 colistin-resistant strains and 198 colistin susceptible strains were correctly detected by the Rapid ResaPolymyxin *Acinetobacter*/*Pseudomonas* NP test. Very major errors (VME) and major errors (ME) corresponded to false-susceptible and false-resistant results, respectively [16]. There were only two ME in *A. baumannii*; details are shown in Tables 1 and 2. The specificity of *A. baumannii* was 96%; while the sensitivity and specificity of the Rapid ResaPolymyxin *Acinetobacter*/*Pseudomonas* NP test to *P. aeruginosa*, *K. pneumoniae* and *E. coli* were 100% (Table 3).

**Table 1:** Colistin MICs obtained by broth microdilution and results of the Rapid ResaPolymyxin *Acinetobacter*/*Pseudomonas* NP test

Isolate	Species	Resistant Phenotype	MIC (mg/L)	Rapid ResaPolymyxin *Acinetobacter*/*Pseudomonas* NP Test	
				Result	Discrepancies with BMD MIC colistin result
BM1539	*A. baumannii*	R	8	Positive	No
BM1579	*A. baumannii*	R	4	Positive	No
BM1595	*A. baumannii*	R	4	Positive	No
BM2349	*A. baumannii*	R	4	Positive	No
BM2370	*A. baumannii*	R	16	Positive	No
BM2412	*A. baumannii*	R	4	Positive	No
BM2431	*A. baumannii*	R	8	Positive	No
BM2622	*A. baumannii*	R	8	Positive	No
TL1671	*P. aeruginosa*	R	4	Positive	No
TL1722	*P. aeruginosa*	R	4	Positive	No
TL1736	*P. aeruginosa*	R	≥32	Positive	No
TL1744	*P. aeruginosa*	R	4	Positive	No
TL2204	*P. aeruginosa*	R	4	Positive	No
TL2294	*P. aeruginosa*	R	4	Positive	No
TL2314	*P. aeruginosa*	R	≥32	Positive	No
TL2917	*P. aeruginosa*	R	4	Positive	No
TL2967	*P. aeruginosa*	R	4	Positive	No
TL3008	*P. aeruginosa*	R	16	Positive	No
TL3086	*P. aeruginosa*	R	≥32	Positive	No
FK20	*K. pneumoniae*	R	≥32	Positive	No
FK26	*K. pneumoniae*	R	≥32	Positive	No
FK150	*K. pneumoniae*	R	≥32	Positive	No
FK169	*K. pneumoniae*	R	≥32	Positive	No
FK171	*K. pneumoniae*	R	≥32	Positive	No
FK591	*K. pneumoniae*	R	≥32	Positive	No
FK610	*K. pneumoniae*	R	≥32	Positive	No
FK1342	*K. pneumoniae*	R	≥32	Positive	No
FK1913	*K. pneumoniae*	R	≥32	Positive	No
FK1986	*K. pneumoniae*	R	8	Positive	No
FK2066	*K. pneumoniae*	R	≥32	Positive	No
FK2166	*K. pneumoniae*	R	≥32	Positive	No
FK2778	*K. pneumoniae*	R	≥32	Positive	No
FK2911	*K. pneumoniae*	R	≥32	Positive	No
FK3789	*K. pneumoniae*	R	≥32	Positive	No
FK3810	*K. pneumoniae*	R	≥32	Positive	No
FK3994	*K. pneumoniae*	R	≥32	Positive	No
FK6556	*K. pneumoniae*	R	32	Positive	No
FK6663	*K. pneumoniae*	R	32	Positive	No
FK6696	*K. pneumoniae*	R	16	Positive	No
DC90	*E. coli*	R	8	Positive	No
DC2562	*E. coli*	R	8	Positive	No
DC3411	*E. coli*	R	4	Positive	No
DC3539	*E. coli*	R	16	Positive	No
DC3599	*E. coli*	R	8	Positive	No
DC3658	*E. coli*	R	8	Positive	No
DC3737	*E. coli*	R	8	Positive	No
DC3802	*E. coli*	R	4	Positive	No
DC3806	*E. coli*	R	8	Positive	No
DC3846	*E. coli*	R	16	Positive	No
DC4887	*E. coli*	R	8	Positive	No
DC5262	*E. coli*	R	8	Positive	No
DC5286	*E. coli*	R	8	Positive	No
DC7333	*E. coli*	R	4	Positive	No
BM1505	*A. baumannii*	S	0.125	Negative	No
BM1506	*A. baumannii*	S	0.5	Negative	No
BM1507	*A. baumannii*	S	0.06	Negative	No
BM1508	*A. baumannii*	S	0.125	Negative	No
BM1509	*A. baumannii*	S	0.125	Negative	No
BM1510	*A. baumannii*	S	0.125	Negative	No
BM1511	*A. baumannii*	S	0.125	Negative	No
BM1512	*A. baumannii*	S	0.25	Negative	No
BM1513	*A. baumannii*	S	0.125	Negative	No
BM1514	*A. baumannii*	S	0.125	Negative	No
BM4151	*A. baumannii*	S	0.25	Negative	No
BM4152	*A. baumannii*	S	0.06	Negative	No
BM4153	*A. baumannii*	S	0.03	Negative	No
BM4154	*A. baumannii*	S	0.125	Negative	No
BM4155	*A. baumannii*	S	0.125	Negative	No
BM4156	*A. baumannii*	S	0.125	Negative	No
BM4158	*A. baumannii*	S	0.125	Negative	No
BM4159	*A. baumannii*	S	0.125	Negative	No
BM4160	*A. baumannii*	S	0.5	Negative	No
BM4161	*A. baumannii*	S	0.125	Negative	No
BM4162	*A. baumannii*	S	0.06	Negative	No
BM4163	*A. baumannii*	S	0.06	Negative	No
BM4164	*A. baumannii*	S	0.06	Negative	No
BM4165	*A. baumannii*	S	0.06	Negative	No
BM4166	*A. baumannii*	S	0.125	Negative	No
BM4167	*A. baumannii*	S	0.125	Negative	No
BM4168	*A. baumannii*	S	0.25	Negative	No
BM4169	*A. baumannii*	S	0.5	Negative	No
BM4170	*A. baumannii*	S	0.125	Negative	No
BM4171	*A. baumannii*	S	0.06	Negative	No
BM4172	*A. baumannii*	S	≤0.06	Negative	No
BM4173	*A. baumannii*	S	0.06	Negative	No
BM4174	*A. baumannii*	S	0.06	Negative	No
BM4175	*A. baumannii*	S	2	Negative	No
BM4176	*A. baumannii*	S	0.06	Negative	No
BM4177	*A. baumannii*	S	0.06	Negative	No
BM4178	*A. baumannii*	S	0.06	Negative	No
BM4179	*A. baumannii*	S	0.25	Negative	No
BM4180	*A. baumannii*	S	0.06	Negative	No
BM4181	*A. baumannii*	S	0.125	Negative	No
BM4182	*A. baumannii*	S	0.25	Negative	No
BM4183	*A. baumannii*	S	1	Negative	No
BM4184	*A. baumannii*	S	1	Positive	Yes, ME
BM4185	*A. baumannii*	S	1	Negative	No
BM4186	*A. baumannii*	S	1	Negative	No
BM4187	*A. baumannii*	S	0.125	Negative	No
BM4188	*A. baumannii*	S	0.5	Positive	Yes, ME
BM4189	*A. baumannii*	S	0.5	Negative	No
BM4190	*A. baumannii*	S	0.125	Negative	No
BM4191	*A. baumannii*	S	0.5	Negative	No
TL2916	*P. aeruginosa*	S	0.125	Negative	No
TL2915	*P. aeruginosa*	S	≤0.06	Negative	No
TL2914	*P. aeruginosa*	S	0.125	Negative	No
TL2913	*P. aeruginosa*	S	0.125	Negative	No
TL2911	*P. aeruginosa*	S	0.125	Negative	No
TL2910	*P. aeruginosa*	S	0.25	Negative	No
TL2908	*P. aeruginosa*	S	0.125	Negative	No
TL2907	*P. aeruginosa*	S	0.5	Negative	No
TL2906	*P. aeruginosa*	S	0.125	Negative	No
TL2905	*P. aeruginosa*	S	0.125	Negative	No
TL2904	*P. aeruginosa*	S	0.125	Negative	No
TL2901	*P. aeruginosa*	S	0.125	Negative	No
TL2899	*P. aeruginosa*	S	0.125	Negative	No
TL2898	*P. aeruginosa*	S	0.125	Negative	No
TL2897	*P. aeruginosa*	S	0.125	Negative	No
TL2895	*P. aeruginosa*	S	0.125	Negative	No
TL2893	*P. aeruginosa*	S	≤0.06	Negative	No
TL2892	*P. aeruginosa*	S	0.125	Negative	No
TL2891	*P. aeruginosa*	S	0.06	Negative	No
TL2890	*P. aeruginosa*	S	0.25	Negative	No
TL2889	*P. aeruginosa*	S	0.5	Negative	No
TL2886	*P. aeruginosa*	S	0.5	Negative	No
TL2885	*P. aeruginosa*	S	0.25	Negative	No
TL2884	*P. aeruginosa*	S	0.25	Negative	No
TL2883	*P. aeruginosa*	S	0.5	Negative	No
TL2882	*P. aeruginosa*	S	0.25	Negative	No
TL2881	*P. aeruginosa*	S	0.25	Negative	No
TL2879	*P. aeruginosa*	S	0.25	Negative	No
TL2878	*P. aeruginosa*	S	0.25	Negative	No
TL2877	*P. aeruginosa*	S	0.25	Negative	No
TL2875	*P. aeruginosa*	S	0.25	Negative	No
TL2874	*P. aeruginosa*	S	0.25	Negative	No
TL2873	*P. aeruginosa*	S	1	Negative	No
TL2872	*P. aeruginosa*	S	0.125	Negative	No
TL2871	*P. aeruginosa*	S	0.25	Negative	No
TL2870	*P. aeruginosa*	S	2	Negative	No
TL2869	*P. aeruginosa*	S	0.125	Negative	No
TL2868	*P. aeruginosa*	S	0.125	Negative	No
TL2867	*P. aeruginosa*	S	0.125	Negative	No
TL2866	*P. aeruginosa*	S	0.125	Negative	No
TL2865	*P. aeruginosa*	S	0.125	Negative	No
TL2864	*P. aeruginosa*	S	0.25	Negative	No
TL2863	*P. aeruginosa*	S	≤0.06	Negative	No
TL2862	*P. aeruginosa*	S	0.125	Negative	No
TL2861	*P. aeruginosa*	S	0.25	Negative	No
TL2858	*P. aeruginosa*	S	0.25	Negative	No
TL2857	*P. aeruginosa*	S	0.25	Negative	No
TL2856	*P. aeruginosa*	S	0.125	Negative	No
TL2855	*P. aeruginosa*	S	0.25	Negative	No
TL2854	*P. aeruginosa*	S	0.125	Negative	No
FK3640	*K. pneumoniae*	S	≤0.06	Negative	No
FK3642	*K. pneumoniae*	S	≤0.06	Negative	No
FK3646	*K. pneumoniae*	S	≤0.06	Negative	No
FK3660	*K. pneumoniae*	S	≤0.06	Negative	No
FK3671	*K. pneumoniae*	S	0.125	Negative	No
FK3686	*K. pneumoniae*	S	0.5	Negative	No
FK3695	*K. pneumoniae*	S	≤0.06	Negative	No
FK3696	*K. pneumoniae*	S	≤0.06	Negative	No
FK3703	*K. pneumoniae*	S	≤0.06	Negative	No
FK3712	*K. pneumoniae*	S	≤0.06	Negative	No
FK3719	*K. pneumoniae*	S	≤0.06	Negative	No
FK3721	*K. pneumoniae*	S	≤0.06	Negative	No
FK3724	*K. pneumoniae*	S	1	Negative	No
FK3727	*K. pneumoniae*	S	0.5	Negative	No
FK3730	*K. pneumoniae*	S	≤0.06	Negative	No
FK3732	*K. pneumoniae*	S	0.25	Negative	No
FK3738	*K. pneumoniae*	S	≤0.06	Negative	No
FK3739	*K. pneumoniae*	S	0.125	Negative	No
FK3740	*K. pneumoniae*	S	≤0.06	Negative	No
FK3741	*K. pneumoniae*	S	≤0.06	Negative	No
FK3745	*K. pneumoniae*	S	0.5	Negative	No
FK3746	*K. pneumoniae*	S	1	Negative	No
FK3749	*K. pneumoniae*	S	≤0.06	Negative	No
FK3758	*K. pneumoniae*	S	≤0.06	Negative	No
FK3764	*K. pneumoniae*	S	≤0.06	Negative	No
FK3767	*K. pneumoniae*	S	≤0.06	Negative	No
FK3771	*K. pneumoniae*	S	0.5	Negative	No
FK3784	*K. pneumoniae*	S	≤0.06	Negative	No
FK3800	*K. pneumoniae*	S	0.5	Negative	No
FK3803	*K. pneumoniae*	S	0.25	Negative	No
FK3813	*K. pneumoniae*	S	≤0.06	Negative	No
FK3817	*K. pneumoniae*	S	≤0.06	Negative	No
FK3824	*K. pneumoniae*	S	0.06	Negative	No
FK3830	*K. pneumoniae*	S	≤0.06	Negative	No
FK3831	*K. pneumoniae*	S	≤0.06	Negative	No
FK3838	*K. pneumoniae*	S	0.5	Negative	No
FK3844	*K. pneumoniae*	S	0.125	Negative	No
FK3853	*K. pneumoniae*	S	0.125	Negative	No
FK3878	*K. pneumoniae*	S	0.25	Negative	No
FK3882	*K. pneumoniae*	S	≤0.06	Negative	No
FK3891	*K. pneumoniae*	S	≤0.06	Negative	No
FK3927	*K. pneumoniae*	S	0.125	Negative	No
FK3938	*K. pneumoniae*	S	0.5	Negative	No
FK3943	*K. pneumoniae*	S	0.06	Negative	No
FK3946	*K. pneumoniae*	S	0.5	Negative	No
FK3989	*K. pneumoniae*	S	≤0.06	Negative	No
FK3990	*K. pneumoniae*	S	1	Negative	No
FK3996	*K. pneumoniae*	S	0.125	Negative	No
FK3999	*K. pneumoniae*	S	≤0.06	Negative	No
FK4002	*K. pneumoniae*	S	≤0.06	Negative	No
DC8640	*E. coli*	S	0.25	Negative	No
DC8641	*E. coli*	S	≤0.06	Negative	No
DC8642	*E. coli*	S	0.125	Negative	No
DC8643	*E. coli*	S	0.125	Negative	No
DC8644	*E. coli*	S	0.5	Negative	No
DC8645	*E. coli*	S	≤0.06	Negative	No
DC8646	*E. coli*	S	≤0.06	Negative	No
DC8647	*E. coli*	S	≤0.06	Negative	No
DC8648	*E. coli*	S	≤0.06	Negative	No
DC8649	*E. coli*	S	0.125	Negative	No
DC8650	*E. coli*	S	0.06	Negative	No
DC8651	*E. coli*	S	0.06	Negative	No
DC8652	*E. coli*	S	0.125	Negative	No
DC8653	*E. coli*	S	0.06	Negative	No
DC8654	*E. coli*	S	0.06	Negative	No
DC8655	*E. coli*	S	0.06	Negative	No
DC8656	*E. coli*	S	0.125	Negative	No
DC8657	*E. coli*	S	≤0.06	Negative	No
DC8658	*E. coli*	S	0.06	Negative	No
DC8659	*E. coli*	S	≤0.06	Negative	No
DC8660	*E. coli*	S	≤0.06	Negative	No
DC8661	*E. coli*	S	≤0.06	Negative	No
DC8663	*E. coli*	S	0.125	Negative	No
DC8664	*E. coli*	S	2	Negative	No
DC8665	*E. coli*	S	≤0.06	Negative	No
DC8666	*E. coli*	S	0.06	Negative	No
DC8667	*E. coli*	S	≤0.06	Negative	No
DC8668	*E. coli*	S	≤0.06	Negative	No
DC8669	*E. coli*	S	≤0.06	Negative	No
DC8670	*E. coli*	S	≤0.06	Negative	No
DC8671	*E. coli*	S	≤0.06	Negative	No
DC8672	*E. coli*	S	≤0.06	Negative	No
DC8673	*E. coli*	S	≤0.06	Negative	No
DC8674	*E. coli*	S	0.06	Negative	No
DC8675	*E. coli*	S	≤0.06	Negative	No
DC8676	*E. coli*	S	≤0.06	Negative	No
DC8677	*E. coli*	S	≤0.06	Negative	No
DC8678	*E. coli*	S	≤0.06	Negative	No
DC8679	*E. coli*	S	≤0.06	Negative	No
DC8680	*E. coli*	S	0.25	Negative	No
DC8681	*E. coli*	S	2	Negative	No
DC8682	*E. coli*	S	0.125	Negative	No
DC8683	*E. coli*	S	≤0.06	Negative	No
DC8684	*E. coli*	S	≤0.06	Negative	No
DC8685	*E. coli*	S	≤0.06	Negative	No
DC8686	*E. coli*	S	0.06	Negative	No
DC8687	*E. coli*	S	0.06	Negative	No
DC8688	*E. coli*	S	0.06	Negative	No
DC8690	*E. coli*	S	0.06	Negative	No
DC8691	*E. coli*	S	0.125	Negative	No

*ME* major error, *S* susceptible, *R* resistant

**Table 2:** Colistin MICs for 253 Gram-negative isolates

Organism	Number of isolates	Colistin MIC (mg/L)									
		≤0.06	0.125	0.25	0.5	1	2	4	8	16	≥32
Total	253	86	56	27	19	8	4	14	13	5	21
*A. baumannii*	58	15	19	5	6	4	1	4	3	1	0
*P. aeruginosa*	61	4	23	17	4	1	1	7	0	1	3
*K. pneumoniae*	70	30	6	3	8	3	0	0	1	1	18
*E. coli*	64	37	8	2	1	0	2	3	9	2	0

**Table 3:** Rapid ResaPolymyxin *Acinetobacter*/*Pseudomonas* NP test results among Gram-negative isolates

Organism	Susceptibility to polymyxins	Resistance mechanism	Isolates	Rapid ResaPolymyxin *Acinetobacter*/*Pseudomonas* NP test	Sensitivity	Specificity
*A. baumannii*	Resistant	Mediated by chromosome^a^	8 (3.16%)	8 positive result	100%	96%
	Susceptible		50 (19.76%)	48 negative results and 2 positive result		
*P. aeruginosa*	Resistant	Mediated by chromosome	11 (4.35%)	11 positive result	100%	100%
	Susceptible		50 (19.76%)	50 negative results		
*K. pneumoniae*	Resistant	Mediated by chromosome^a^	20 (7.91%)	20 positive result	100%	100%
	Susceptible		50 (19.76%)	50 negative results		
*E. coli*	Resistant	Mediated by plasmid	14 (5.54%)	2 positive result	100%	100%
	Susceptible		50 (19.76%)	50 negative results		

^a^Unpublished

## Discussion

In this study, we described the diagnostic performance of the Rapid ResaPolymyxin *Acinetobacter*/*Pseudomonas* NP test, a phenotypic method for differentiation between colistin-resistant strains and colistin-susceptible strains. Compared with the reference BMD, the Rapid ResaPolymyxin *Acinetobacter*/*Pseudomonas* NP test showed accuracy in detecting the resistance to colistin. Besides, the method was fast, easy to perform, and the obtained data were easy to interpret. Rapid Polymyxin NP test makes up for the limitations of applicability in non-fermenters [14]. In our study, we examined it efficiency in detecting non-fermentative bacteria, but also fermentative bacteria, such as *E. coli* strains and *K. pneumoniae* strains. The results showed that the sensitivity and specificity of the Rapid ResaPolymyxin *Acinetobacter*/*Pseudomonas* NP test to *Enterobacteriaceae* were 100%, which was consistent with a previous study [15]. In the present study, there were only two ME in colistin-susceptible *A. baumannii* strains. The categorical agreement for all tested isolates was 99.2% for the Rapid ResaPolymyxin *Acinetobacter*/*Pseudomonas* NP test. In addition, the sensitivity and specificity were respectively 100 and 99%, which further suggested that this method is suitable for detecting fermentative bacteria.

So far, a number of studies have examined the mechanism of colistin resistance [17, 18]. This study revealed that chromosome mutations of two-component regulatory systems (TCSs) and *mcr-1*, which were located in plasmid, were the main causes of colistin resistance in 53 strains. In addition, we were able to detect drug resistance without a difference. Therefore, compared with the Rapid Polymyxin NP test, the Rapid ResaPolymyxin *Acinetobacter*/*Pseudomonas* NP test is suitable to be used in more scenes.

MicroScan Colistin Well is a newly developed kit for detection of colistin resistance in Gram-negative bacteria [19]. The fundamental principle of the Rapid ResaPolymyxin *Acinetobacter*/*Pseudomonas* NP test is similar to MicroScan Colistin Well. Both methods can be used to detect living bacteria in the medium with 4 mg/L or 3.75 mg/L of colistin (close to the breakpoint of colistin resistance). Similarly, the MICs cannot be determined utilizing the Rapid ResaPolymyxin *Acinetobacter*/*Pseudomonas* NP test and the MicroScan Colistin Well. Only colistin resistance results or sensitive test results can be obtained by them. However, there are two major differences between the two methods. First, Rapid ResaPolymyxin *Acinetobacter*/*Pseudomonas* NP test is significantly faster compared to MicroScan Colistin Well. For example, the detection of *P. aeruginosa* by Rapid ResaPolymyxin *Acinetobacter*/*Pseudomonas* NP test takes maximum 5 h to analyze the results, while MicroScan Colistin Well requires 16 to 18 h. Secondly, in the presence of resazurin reagent PrestoBlue®, the growth of living bacteria of the Rapid ResaPolymyxin *Acinetobacter*/*Pseudomonas* NP test can be more clearly observed compared to MicroScan Colistin Well.

The principle of Rapid ResaPolymyxin *Acinetobacter*/*Pseudomonas* NP test is based on the visual detection of the reduction of the resazurin reagent, a viability colorant that is observed by color change (blue to purple or pink). Interestingly, in the current study, no significant color changes were observed in colistin-resistant *P. aeruginosa* after the addition of the resazurin reagent for 1 h. After prolonging the observation time for another 1 h, the color changed from blue to purple. In other words, the results were not obtained until 2 h later in the study, while very obvious color changes were observed 15 min after the addition of the resazurin reagent in the colistin-resistant strains of *A. baumanii*, *K. pneumoniae* and *E. coli*, including 2 ME. This may be because the growth rate of *P. aeruginosa* is slower than that of *Enterobacteriaceae*, thus taking longer to decompose resazurin into fluorescent substance resorufin. It suggested that the observation time of the results of this experiment needed to be optimized according to the strain.

However, the Rapid ResaPolymyxin *Acinetobacter*/*Pseudomonas* NP test still has some limitations. Firstly, the accurate MIC values could not be obtained. Since the Rapid ResaPolymyxin *Acinetobacter*/*Pseudomonas* NP test was not suitable for the study of high-level drug resistant strains, the method could only show whether the colistin resistant was present or not. Secondly, several *mcr*-harboring isolates with an MIC of 2 mg/L (or even less) to colistin or polymyxin B have been reported [20, 21], while our method could only be used to screen colistin resistant strains with MIC ≥4 mg/L. Thirdly, the reading time of *P. aeruginosa* results was different from that reported by the inventors, requiring an additional 1 h of observation time.

## Conclusion

The Rapid ResaPolymyxin *Acinetobacter*/*Pseudomonas* NP test has great stability and sensitivity in detection of colistin resistance in Gram-negative bacteria such as *A. baumanii*, *P. aeruginosa*, *K. pneumoniae* and *E. coli* strains. In addition, this method is fast and easy to perform. It can contribute in selecting more precise therapeutic choices, and optimizing antibiotic stewardship, and preventing the development of outbreaks with multidrug-resistant isolates. Nevertheless, the testing time of *P. aeruginosa* is longer than that reported by the inventor, so the observation time of this method needs to be further optimized.

## Methods

### Bacterial strains

A total of 253 nonduplicate clinical Gram-negative isolates including *A. baumanii* strains (*n* = 58), *P. aeruginosa* strains (*n* = 61), *K. pneumoniae* strains (*n* = 70) and *E. coli* strains (*n* = 64) were obtained from a teaching hospital in Wenzhou, China. Species identification was performed using the Matrix-Assisted Laser Desorption Ionization Time-Of-Flight Mass Spectrometry (MALDI-TOF MS, Bruker Daltonics, US). A total of 53 colistin-resistant strains were selected from our previous studies and were detected by BMD, including 8 *A. baumanii* strains, 11 *P. aeruginosa* strains, 20 *K. pneumoniae* strains and 14 *E. coli* strains. In addition, 50 colistin-susceptible isolates of each four bacterial species mentioned above were randomly selected as the control group. *E. coli* ATCC 25922 and *P. aeruginosa* ATCC 27853 were used as control strains [6].

### Antimicrobial susceptibility test

BMD was performed in triplicate. According to the EUCAST/CLSI joined guidelines [6, 7], the clinical breakpoints for colistin provided for *P. aeruginosa* and *A. baumanii* were ≤ 2 mg/L (susceptible breakpoint) and ≥ 4 mg/L (resistant breakpoint) and *Enterobacteriaceae* are ≤2 mg/L (susceptible breakpoint) and > 2 mg/L (resistant breakpoint).

### Rapid ResaPolymyxin *Acinetobacter*/*Pseudomonas* NP test

The experimental procedure was performed according to the previously described protocol [15]. Briefly, the colistin-containing Mueller Hinton broth (MHB, OXOID, UK) solution was prepared with an initial concentration of 4.16 mg/L. Then, a 180 μl colistin-free MHB solution and colistin-containing MHB solution were added to lines A and B of a 96-well polystyrene micro test plate, respectively. For each isolate, 20 μl of the bacterial suspension at a 3.5 McFarland optical density (~ 1 × 10^9^ CFU/mL) was inoculated in parallel into two wells, with and without colistin. The bacterial suspension was mixed with the medium by pipetting up and down. The final concentration of colistin was 3.75 mg/L. In the same way, 20 μl of 0.85% NaCl was used as an aseptic control, 20 μl of the colistin-susceptible isolate (*E. coli* ATCC 25922 and *P. aeruginosa* ATCC 27853) suspension was used as negative control; and 20 μl of the colistin-resistant isolate (the clinical isolates of Morgan, inherent resistance to polymyxin) suspension was used as a positive control. After testing several isolates, we ensured that the color-transfer of colistin suspension and the mixing of bacterial suspension in the micro test plate were completed within 15 min. The inoculated tray was incubated at 35 ± 2 °C for 3 h. Then, 22 μl of the resazurin reagent PrestoBlue® (ThermoFisher Scientific, US, final concentration is 10% V/V) was added per well and each well was mixed by pipetting up and down. Finally, the tray was visually inspected every 15 min within 1 h. Susceptibility of colistin is determined by the color changes, where discoloration indicates that the strain is colistin-resistant, while the lack of discoloration indicates that the strain is colistin-susceptible [15]. All experiments were performed in triplicate.

**Fig. 1:**
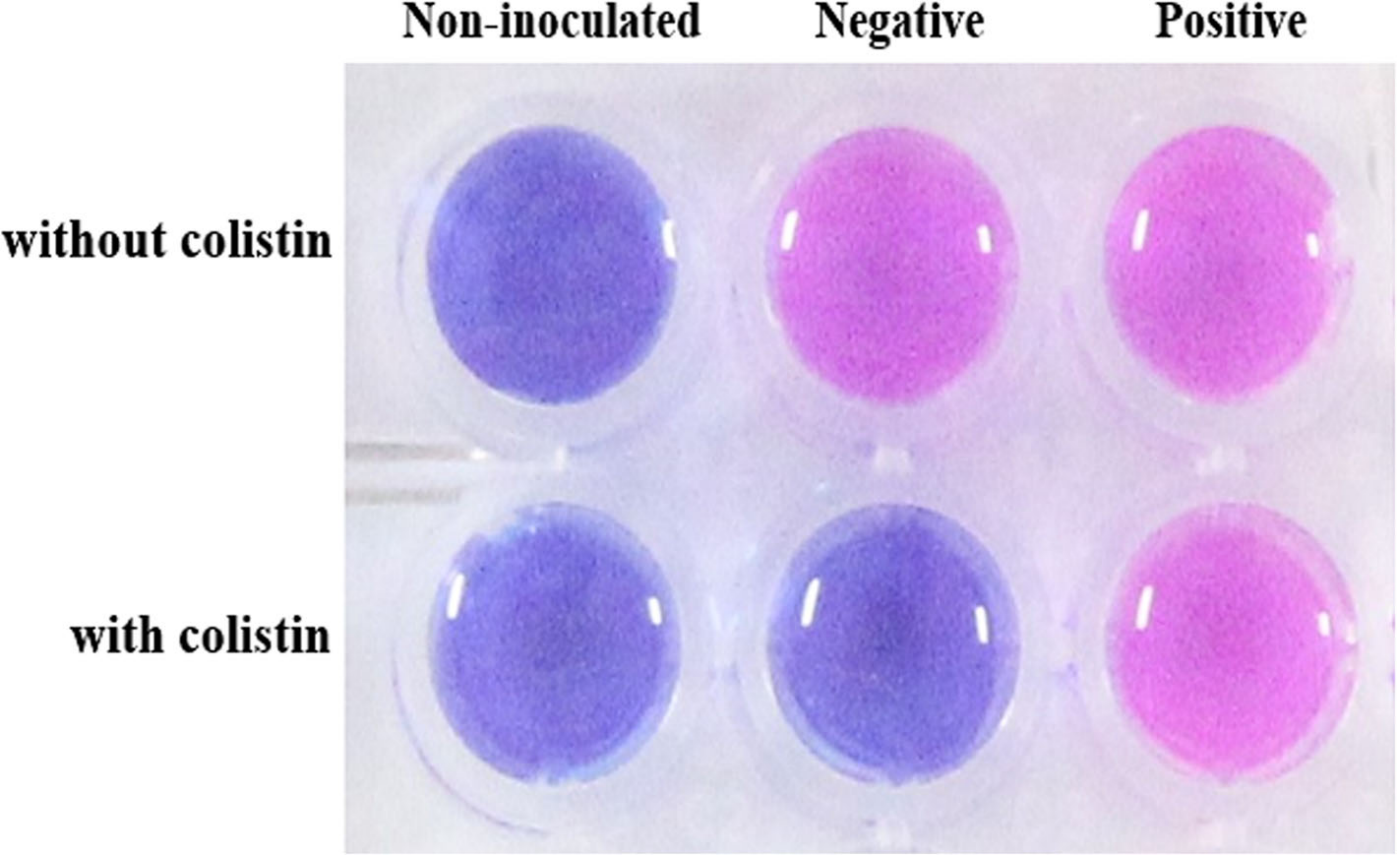
Representative results of the Rapid ResaPolymyxin *Acinetobacter*/*Pseudomonas* NP test. Non-inoculated well is shown as the control of the medium and the color changed (first column). Negative, the tested isolate only grows in the absence of colistin (second column). Positive, the tested isolate grows in the presence and absence of colistin (third column)

The test was considered to be positive (i.e., purple or pink) if the colistin-resistant isolate was viable in presence of colistin, or negative (i.e., blue) if the colistin-susceptible isolate was not viable in presence of colistin. The Rapid ResaPolymyxin *Acinetobacter*/*Pseudomonas* NP test interpretation is illustrated in Fig. 1.

## Data Availability

All data generated or analyzed during this study are included in this published article.

## Abbreviations

ATCC: American Type Cultures Collection
BMD: Broth Microdilution
CFU: Colony Forming Unit
CLSI: Clinical and Laboratory Standards Institute
EUCAST: European Committee of Antibiotic Susceptibility Testing
MDR: Multidrug resistance
ME: Major Error
MHB: Mueller Hinton broth
MIC: Minimal Inhibitory Concentration
VME: Very Major Error
